# HAlign 4: a new strategy for rapidly aligning millions of sequences

**DOI:** 10.1093/bioinformatics/btae718

**Published:** 2024-11-28

**Authors:** Tong Zhou, Pinglu Zhang, Quan Zou, Wu Han

**Affiliations:** Institute of Fundamental and Frontier Sciences, University of Electronic Science and Technology of China, Chengdu, Sichuan 610054, China; Yangtze Delta Region Institute (Quzhou), University of Electronic Science and Technology of China, Quzhou, Zhejiang 324003, China; Institute of Fundamental and Frontier Sciences, University of Electronic Science and Technology of China, Chengdu, Sichuan 610054, China; Yangtze Delta Region Institute (Quzhou), University of Electronic Science and Technology of China, Quzhou, Zhejiang 324003, China; Institute of Fundamental and Frontier Sciences, University of Electronic Science and Technology of China, Chengdu, Sichuan 610054, China; Yangtze Delta Region Institute (Quzhou), University of Electronic Science and Technology of China, Quzhou, Zhejiang 324003, China; Department of Statistics, Stanford University, Stanford, CA 94305-4065, United States

## Abstract

**Motivation:**

HAlign is a high-performance multiple sequence alignment software based on the star alignment strategy, which is the preferred choice for rapidly aligning large numbers of sequences. HAlign3, implemented in Java, is the latest version capable of aligning an ultra-large number of similar DNA/RNA sequences. However, HAlign3 still struggles with long sequences and extremely large numbers of sequences.

**Results:**

To address this issue, we have implemented HAlign4 in C++. In this version, we replaced the original suffix tree with Burrows–Wheeler Transform and introduced the wavefront alignment algorithm to further optimize both time and memory efficiency. Experiments show that HAlign4 significantly outperforms HAlign3 in runtime and memory usage in both single-threaded and multi-threaded configurations, while maintains high alignment accuracy comparable to MAFFT. HAlign4 can complete the alignment of 10 million coronavirus disease 2019 (COVID-19) sequences in about 12 min and 300 GB of memory using 96 threads, demonstrating its efficiency and practicality for large-scale alignment on standard workstations.

**Availability and implementation:**

Source code is available at https://github.com/malabz/HAlign-4, dataset is available at https://zenodo.org/records/13934503.

## 1 Introduction

Multiple sequence alignment (MSA) is crucial in bioinformatics for analyzing biological sequence structures, functions, and phylogenetic inferences (Edgar and Batzoglou 2006, Tian *et al.* 2024). Over the last decade, the costs of next-generation sequencing have halved each year, outpacing the reduction in computational costs. This significant progress has led to the generation of hundreds of thousands of metagenomes and billions of putative gene sequences (Markowitz *et al.* 2014, Wilke *et al.* 2016). Given that MSA is an NP-hard problem (Wang and Jiang 1994), aligning such large-scale sequence data (ranging from millions to tens of millions of sequences) has become an urgent issue that needs to be addressed. In recent years, advancements in sequence alignment (Marco-Sola *et al.* 2021, Marco-Sola *et al.* 2023, Zhai, Chao *et al.* 2024, Zhang *et al.* 2024) and parallel computing have made it feasible to align ultra-large-scale sequence data (Garriga *et al.* 2019).

HAlign is a multiple sequence alignment software implemented in Java, based on the center star strategy (Zou *et al.* 2015). The primary workflow of HAlign involves selecting the longest sequence among all sequences as the central sequence and constructing a suffix tree for it to identify common substrings during pairwise alignment. Subsequently, all remaining sequences are aligned with the central sequence in a pairwise manner. This process is particularly well-suited for acceleration using parallel computing techniques. HAlign (Zou *et al.* 2015) utilizes Hadoop, HAlign2 (Wan and Zou 2017) uses Spark, and HAlign3 (Tang *et al.* 2022) leverages multithreading parallel techniques to accelerate MSA. Finally, all pairwise alignments are merged into a multiple sequence alignment using the central sequence as a bridge. The HAlign series excels at rapidly aligning large numbers of similar nucleotide sequences, with HAlign3 capable of aligning 1 million Severe acute respiratory syndrome coronavirus 2 (SARS-CoV-2) genomes in 30 min (Tang *et al.* 2022).

The latest version, HAlign3, utilizes a left-child right-sibling (LCRS) tree to construct a suffix tree (Ukkonen 1995) for the central star sequence, enabling the identification of common substrings in pairwise sequences. For the remaining segments, it uses affine gap penalty and k-banded dynamic programming for alignment. By adopting these techniques, HAlign3 efficiently aligns vast numbers of similar DNA/RNA sequences, addressing the growing challenge of intraspecific sequence accumulation driven by advancements in sequencing technology. However, when the sequence data is long, constructing a suffix tree consumes a significant amount of memory, and the resulting long segments can cause dynamic programming to overflow. In addition, when the number of sequences reaches millions or even tens of millions, the memory requirements of HAlign3 exceed the capacity of standard workstations, making it challenging to support.

To address these issues, we implement HAlign4 in C++ and make two major improvements. In HAlign4, we replace the suffix tree used in HAlign3 with Burrows–Wheeler Transform (BWT) (Michael Burrows 1994) for the central sequence, which accelerates the search for common substrings. For the alignment of subsequent segments, we substitute the original affine gap penalty and k-banded dynamic programming with the wavefront alignment algorithm (Marco-Sola *et al.* 2021, Marco-Sola *et al.* 2023). This modification is made because the wavefront alignment algorithm can handle extremely long sequences and, with a time complexity of O(ns) (where n is the sequence length and is the alignment score), it offers very fast alignment speeds for highly similar sequences.

HAlign4 represents a significant advancement in the field of multiple sequence alignment, addressing the key challenges of scalability and memory efficiency. By implementing BWT and the wavefront alignment algorithm, HAlign4 provides a powerful tool for researchers, enabling the alignment of ultra-large sequence datasets using standard computational resources. These enhancements ensure that HAlign4 meets the growing demands of modern genomic research, making large-scale MSA more accessible and practical.

## 2 Materials and methods

### 2.1 Overview of HAlign4

**Figure 1.**
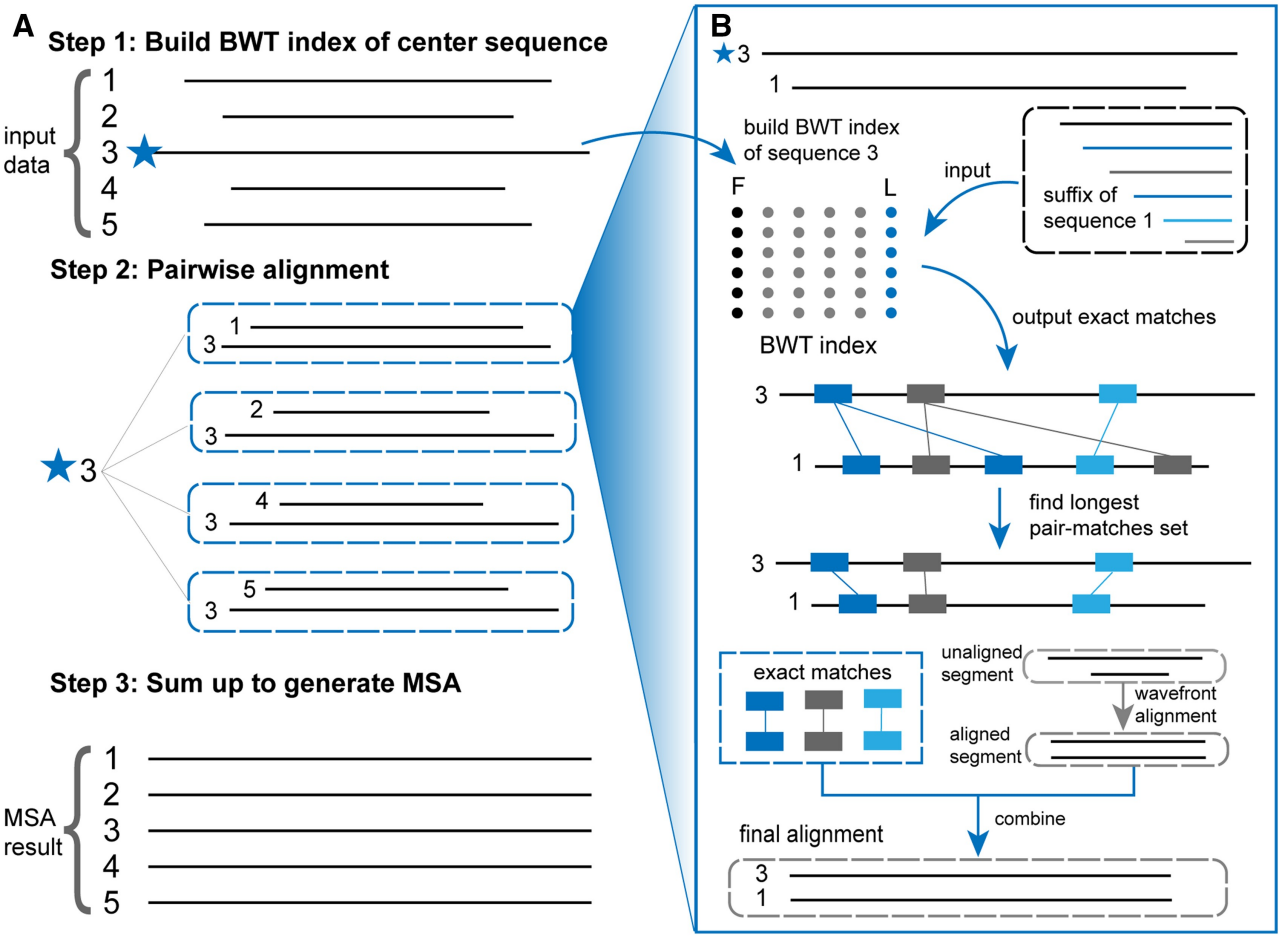
The overall workflow consists of three main steps: (A) the first step is to build the Burrows–Wheeler Transform (BWT) index of the center sequence, which serves as the reference for alignment. The second step involves pairwise alignment of the central sequence with each input sequence using the BWT index. Exact matches are identified, and the longest pair-matches set is found. The third step combines the pairwise alignments to generate the final multiple sequence alignment (MSA). (B) The detailed process of pairwise alignment includes constructing the BWT index for the central sequence (sequence 3), inputting the suffix of another sequence (sequence 1) to find exact matches using the BWT index, and identifying the longest set of pair-matches between the central sequence and the query sequence. For unaligned segments, the wavefront alignment algorithm is used to perform pairwise alignment, enhancing the alignment of long sequences. Finally, the aligned segments are integrated to obtain the final alignment between the sequences, resulting in a comprehensive MSA that leverages the efficiency of BWT and the accuracy of wavefront alignment.

HAlign4 comprises three primary stages, as illustrated in Fig. 1. The first stage involves reading the input data, selecting the central sequence, and constructing the BWT index. Initially, the sequence data is read and preprocessed to ensure integrity, such as replacing ambiguous nucleotides (“N”) with random bases. The longest sequence is then chosen as the central sequence because it is likely to contain the most genetic information, enabling the identification of a greater number of common substrings with the other sequences. This central sequence is then used to construct the BWT index. The second stage focuses on pairwise alignment of all remaining sequences against this central sequence. The BWT index facilitates the identification of common substrings between sequences, followed by dynamic programming to select appropriate substrings for segmentation. Each unaligned segment is subsequently aligned using the wavefront alignment algorithm (WFA). This stage also uses multithreading to accelerate alignment by identifying almost-unique exact matches (MAM) (Marçais *et al.* 2018) between the central sequence and the query sequences. After sorting the pair-matches, dynamic programming is used to select the longest set of pair-matches for segmenting the sequences. The enhancements in HAlign4, compared to HAlign3, are focused on improving this second stage. In the final stage, all pairwise alignment results are merged into a multiple sequence alignment, using the central sequence as the reference.

### 2.2 BWT index construction

In HAlign4, we replace the suffix tree used in HAlign3 with BWT index to search for common substrings. The BWT (Michael Burrows 1994), also known as block-sorting compression, rearranges a character string into runs of similar characters, facilitating compression techniques like move-to-front transform and run-length encoding. Importantly, BWT is reversible and only requires the position of the first original character to be stored that is useful for efficient substring searches. In contrast, a suffix tree is a compressed trie of all the suffixes of a given string, allowing for efficient pattern matching and substring search operations. Both BWTs and suffix trees are crucial data structures in bioinformatics, possessing powerful capabilities for addressing string-related problems. While both suffix tree and BWT require linear memory to represent the center sequence, carefully implemented BWTs have smaller constant factors, thereby reducing the overall memory usage. Given that BWTs can also efficiently query the longest common substrings, we replace the suffix tree with a BWT in this update. BWT can be efficiently implemented using suffix array, achieving linear time complexity. We utilize divsufsort to construct the suffix array of the central star sequence in linear time.

### 2.3 Common substrings searching

During pairwise alignment between the central sequence and other sequences, it is crucial to identify homologous regions for segmenting the sequences, similar to the approach used in MUMmer (Marçais *et al.* 2018). We establish a minimum length threshold, l, and search for the longest common substring at each position in the query sequence. For each suffix of the query sequence, a backward search on the BWT is performed, iterating from the end to the beginning while narrowing the possible match range at each step. This process continues until the entire suffix is matched or no further matches are found, enabling efficient identification of maximal matching substrings. If a substring$\;\left(x,\;y,\;w\right)$ is found, where x is the start position in the central sequence, y is the start position in the query sequence, and w is the length of the substring, with w > l, it is considered a candidate homologous region. The search for the next candidate begins at x+w-l+1.

### 2.4 Select longest pair-matches

The initial step in selecting the longest pair-matches involves sorting them. We utilize topological sorting (Kahn 1962) to establish the preliminary order of pair-matches. We treat each pair-match as a node in a graph. If the ending positions of pair-match i in both sequences are less than the ending positions of pair-match j, we draw a directed edge from i to j. The weight of this edge is the length of match j minus the overlap length between matches i and j. After adding edges between each pair of nodes, we obtain a weighted directed graph. By performing a topological sort on the nodes of this graph, we can determine the preliminary order of the pair-matches. We then use a variant of the longest increasing subsequence algorithm to find the longest set of pair-matches that occur in ascending order without overlap in both the central sequence and the query sequence.

### 2.5 Wavefront alignment

After segmenting the two sequences based on the final pair-matches, each segment requires pairwise alignment. Wavefront alignment is a recently developed method that leverages homologous regions between sequences to expedite the alignment process. It operates in O(ns) time, where n is the read length and s is the alignment score. This efficiency makes wavefront alignment substantially faster than traditional dynamic programming methods, especially when dealing with long and highly similar sequences. In practice, we configure the dynamic programming parameters as follows: affine gap penalties with a match score of 0, a mismatch penalty of 2, a gap opening penalty of 3, and a gap extension penalty of 1. The HAlign series aims to provide rapid alignment solutions for highly similar nucleotide sequences. The integration of the WFA into the HAlign workflow not only accelerates the alignment process but also enhances the capability to accurately align long sequences.

## 3 Results

The experiments are conducted on a system running Ubuntu 16.04.7 LTS, equipped with an Intel Xeon Platinum 8168 CPU at 2.70 GHz, 96 cores, and approximately 1 TB of memory. HAlign4, an open-source C++ implementation, is extensively tested to evaluate its performance in terms of runtime, memory usage, and alignment accuracy. These tests involve comparisons with various established methods on both simulated and real datasets. The methods included in the study are MAFFT (Katoh *et al.* 2002), HAlign4, HAlign3 (Tang *et al.* 2022), MUSCLE3, and ClustalΩ (Sievers and Higgins 2014).

### 3.1 Dataset

To conduct the comparisons, we utilize both simulated and real sequence data. For the simulated datasets, we use INDEible (Fletcher and Yang 2009) to generate mitochondrial-like sequences with similarity levels ranging from 70% to 99%, based on parameters used in HAlign3. For datasets of varying lengths, we use the complete monkeypox virus genome (Ma *et al.* 2022) as a template, selecting the longest 1000 sequences and truncating them to lengths ranging from 10 to 100 000 bases. This allowe us to generate simulated datasets of different sequence lengths. For real datasets, we use the mt1x dataset from mtDB (Ingman and Gyllensten 2006), as well as three SARS-CoV-2 datasets. The mt1x dataset contains 672 sequences with an average length of 16 568 bases, while the SARS-CoV-2 datasets have an average sequence length of 29 774 bases, and are divided into sets of 500 sequences, 1 million sequences, and 10 million sequences. The 10 million data points are generated by replicating the 1 million data points ten times.

### 3.2 Evaluation metrics

Sequence alignment quality is assessed using several benchmark metrics, including the Sum-of-Pairs (SP) (Altschul 1989) score, the Q score (Edgar 2004), and the Total Column (TC) score. The SP score evaluates the alignment by summing the scores of all sequence pairs, with matches assigned a score of 1, mismatches −1, and gaps −2; lower SP scores indicate higher alignment precision. The scaled SP score (Scaled-SP) normalizes the SP score based on sequence length, facilitating comparisons across different alignments, as shown in formula 1:


(1) scaled SP=2*SPn*n−1*L,

where the SP is the total score of the sequence alignment, *n* is the number of sequences, and *L* is the length of the aligned sequences. The Q score ranges from 0 to 1 and is used to compare a multiple sequence alignment to a reference, with a value of 1 indicating a perfect match. The TC score, which measures only correctly aligned columns against a reference, is stricter than both the SP and Q scores. Collectively, these metrics provide a comprehensive assessment of alignment quality.

### 3.3 Comparison of HAlign4 and HAlign3 on simulated datasets

**Figure 2.**
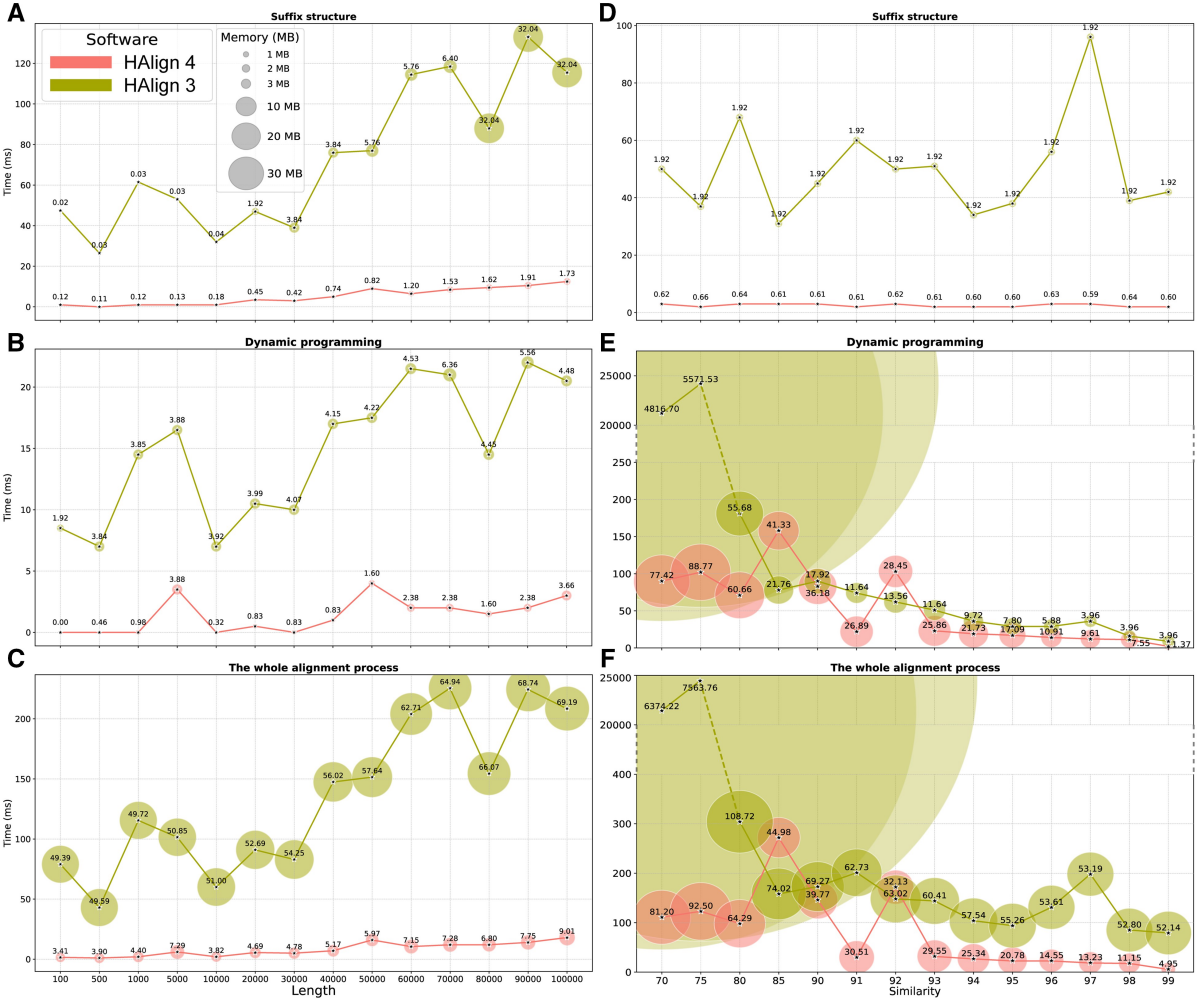
Performance comparison between HAlign 4and HAlign 3. (A–C) Time and memory usage for suffix structure construction, dynamic programming, and the whole alignment process across different sequence lengths. (D–F) Time and memory usage for suffix structure construction, dynamic programming, and the whole alignment process across different sequence similarities. Circles represent memory usage (in MB). The horizontal axis indicates sequence length or similarity, and the vertical axis indicates time (in ms).

We conducte a comprehensive comparison between HAlign4 and HAlign3 on simulated datasets of varying lengths and similarities, as depicted in Fig. 2. The results clearly demonstrate that HAlign4 consistently surpasses HAlign3 in both time and memory efficiency.

In Fig. 2A–C, which illustrate performance across different sequence lengths, HAlign4 shows significant advantages. For suffix structure construction (Fig. 2A), HAlign4 exhibits markedly lower memory consumption and faster execution times compared to HAlign3. During dynamic programming (Fig. 2B), HAlign4 continues to outperform, requiring less time and memory. The whole alignment process (Fig. 2C) further confirms HAlign4’s superiority, as it consistently uses less memory and completes the alignment process more quickly than HAlign3.

Figure 2D–F focus on performance across different sequence similarities. In suffix structure construction (Fig. 2D), HAlign4 maintains its efficiency, with lower memory usage and faster execution times, irrespective of sequence similarity. The benefits of HAlign4 are especially pronounced in dynamic programming at higher sequence similarities (Fig. 2E), where it significantly outperforms HAlign3. The whole alignment process (Fig. 2F) reiterates HAlign4’s robustness and efficiency across varying levels of sequence similarity, highlighting its consistent performance advantage.

### 3.4 Comparison of HAlign4 and other methods on simulated datasets

**Figure 3.**
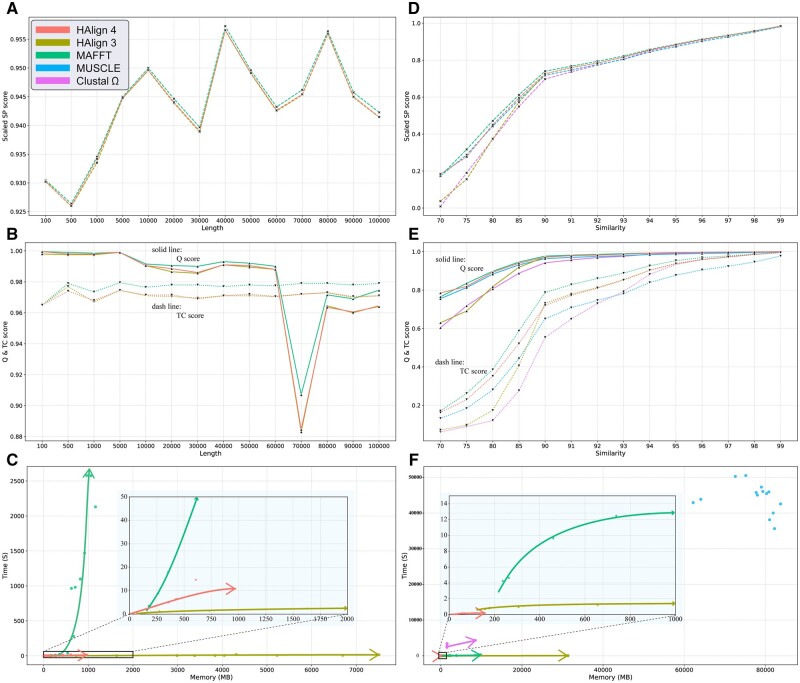
Performance and accuracy comparison of HAlign4, HAlign3, MAFFT, MUSCLE, and Clustal Omega. (A–C) show scaled SP score, Q score (solid line), TC score (dashed line), and time and memory usage across different sequence lengths. (D–F) display the same metrics across different sequence similarities. Horizontal axes in (A–C) represent sequence length, while in (D–F) they represent sequence similarity. Vertical axes in (A, D) represent scaled SP score, in (B, E) Q and TC scores, and in (C, F) time (s) and memory (MB). *Note*: For a detailed explanation of the SP score, Q score, and TC score metrics, please refer to Section 3.2.

Figure 3 illustrates a comprehensive performance and accuracy comparison of HAlign 4, HAlign 3, MAFFT, MUSCLE, and ClustalΩ on simulated datasets, focusing on several critical metrics including scaled SP score, Q score, TC score, time, and memory usage across various sequence lengths and similarities.

Figure 3A and B shows the comparison of alignment quality of various MSA methods across different sequence lengths. It can be observed that MUSCLE and ClustalΩ fail to complete alignments for longer sequences, while MAFFT achieves higher alignment quality than other methods. The difference between HAlign4 and HAlign3 is minimal, with both slightly trailing behind MAFFT. Figure 3C illustrates the time and memory usage trends of different methods as sequence length increases. MAFFT’s alignment time significantly increases with longer sequences, and HAlign3 shows a substantial increase in memory usage with sequence length. In contrast, HAlign4 demonstrates high robustness in both time and memory efficiency.

On the other hand, we also explore the trends in time, memory, and similarity with different methods as sequence similarity changes. Figure 3D and E shows the changes in alignment quality. It can be observed that MAFFT consistently achieves the highest alignment quality, followed by HAlign4. MUSCLE and ClustalΩ exhibit the poorest alignment quality. HAlign3 has very low alignment quality for low-similarity sequences, but its quality gradually approaches that of HAlign4 as sequence similarity increases. Regarding alignment time and memory usage, MUSCLE and ClustalΩ perform poorly, especially MUSCLE. For the other three methods, as sequence similarity decreases, both alignment time and memory usage increase. MAFFT shows the most significant increase, followed by HAlign3, while HAlign4 maintains exceptional performance with minimal increases in time and memory usage.

### 3.5 Comparison of HAlign4 and other methods on real datasets

We also compare the performance of HAlign4, HAlign3, MAFFT, MUSCLE, and ClustalΩ on real datasets to evaluate their efficiency and accuracy under practical conditions. The results, summarized in Tables 1 and 2, highlight significant differences in alignment time, memory usage, and accuracy among the methods. The result for MUSCLE was not displayed because the tool failed to complete the alignment within a reasonable time and memory constraint.

**Table 1.** Performance comparison for mt1x dataset.^a^

Software	t1-time	t1-mem	t8-time	t8-mem	Scaled-SP
HAlign4	**0.4 s**	**17.4 MB**	**0.2 s**	**19.8 MB**	0.991525
HAlign3	1.9 s	300.5 MB	1.7 s	325.2 MB	**0.991586**
MAFFT	2 min 19 s	148.3 MB	25.6 s	632.8 MB	0.991043
ClustalΩ	6 h 44 min 7 s	1.7 GB	5 h 31 min 28 s	1.7 GB	0.991295

a The first row lists the alignment software used in the comparison: HAlign4, HAlign3, MAFFT, MUSCLE, and ClustalΩ. Columns indicate: t1-time (single-threaded alignment time), t1-mem (single-threaded memory usage), t8-time (8-thread alignment time), t8-mem (8-thread memory usage), and Scaled-SP (Scaled Sum-of-Pairs alignment accuracy score). Bold values represent the best-performing results in each column.

**Table 2.** Performance comparison for SARS-CoV-2-500 dataset.^a^

Software	t1-time	t1-mem	t8-time	t8-mem	Scaled-SP
HAlign4	**0.5 s**	**22.8 MB**	**0.2 s**	**29.6 MB**	0.98666
HAlign3	2 s	407.0 MB	1.9 s	405.2 MB	0.98666
MAFFT	2 min 59 s	170.8 MB	46.8 s	528.2 MB	**0.98676**
ClustalΩ	16 h 2 min 32 s	5.2 GB	16 h 5 min 23 s	5.3 GB	0.98673

a The remaining details are consistent with those provided in Table 1.

Table 1 presents the performance metrics for the mt1x dataset. HAlign4 demonstrates remarkable efficiency with a single-threaded alignment time of 0.4 s and memory usage of 17.4 MB. Its multi-threaded performance was equally impressive, completing the alignment in 0.2 s with 19.8 MB of memory. The Scaled-SP score of 0.991525 indicates high alignment accuracy. HAlign3, while accurate (Scaled-SP score of 0.991586), required significantly more resources, taking 1.9 s and 300.5 MB of memory for single-threaded alignment and 1.7 s and 325.2 MB for multi-threaded alignment. MAFFT shows good alignment quality (Scaled-SP score of 0.991043) but is less efficient, particularly in multi-threaded mode, where it requires 25.6 s and 632.8 MB of memory. MUSCLE fails to complete the alignment within reasonable time and memory limits, with ClustalΩ showing the longest times and highest memory usage.

Table 2 illustrates the performance on the SARS-CoV-2-500 dataset. Again, HAlign4 outperforms other methods with a single-threaded alignment time of 0.5 s and memory usage of 22.8 MB. Its multi-threaded performance remained strong, completing in 0.2 s with 29.6 MB of memory. The Scaled-SP score is slightly lower at 0.98666, but still indicative of high alignment accuracy. HAlign3, while accurate (Scaled-SP score of 0.98666), shows higher resource consumption, taking 2 s and 407.0 MB of memory for single-threaded alignment and 1.9 s and 405.2 MB for multi-threaded alignment. MAFFT, despite good alignment quality (Scaled-SP score of 0.98676), required much longer times, especially in multi-threaded mode (46.8 s and 528.2 MB of memory). MUSCLE again fails to complete the alignment efficiently, with ClustalΩ showing extreme times and memory usage.

We also conduct experiments on COVID-19 datasets consisting of 1 million and 10 million sequences, utilizing 96 threads for both HAlign3 and HAlign4. For the 1 million sequence dataset, HAlign3 requires 12 min and 53 s with 505 GB of memory, whereas HAlign4 completes the task in just 2 min and 26 s, using only 32 GB of memory. When aligning the 10 million sequence dataset, HAlign3 is unable to complete the task, while HAlign4 successfully finishes in 26 min and 15 s, using 318 GB of memory. These results highlight HAlign4’s significant memory optimization, making large-scale multiple sequence alignments feasible on more typical workstations.

## 4 Discussion

In this study, we introduce HAlign4, a substantial advancement over its predecessor HAlign3, designed to tackle the challenges inherent in large-scale multiple sequence alignment. Our findings indicate that HAlign4 consistently surpasses HAlign3 in both computational efficiency and memory usage across a wide range of simulated and real-world datasets. Notably, HAlign4 incorporates significant algorithmic enhancements, including the BWT index and wavefront alignment, which collectively contribute to its superior performance. These optimizations allow HAlign4 to handle larger datasets with enhanced efficiency, reducing computational demands and enabling its use on standard workstations without the need for specialized hardware infrastructure. The implementation in C++ further contributes to its optimized performance, allowing researchers to efficiently utilize available resources and achieve rapid alignment results even for ultra-large datasets.

The experiments conducted on COVID-19 datasets further demonstrate the scalability of HAlign4. For example, when aligning a dataset of 1 million sequences, HAlign3 requires 505 GB of memory, while HAlign4 completes the alignment using only 32 GB of memory. In contrast to HAlign3, which faces significant memory constraints and fails to complete the alignment of the largest dataset, HAlign4 exhibits robust performance, completing the alignment in a fraction of the time and with considerably lower memory consumption. This ability to effectively manage large-scale datasets is crucial in contemporary bioinformatics, where the volume of sequence data is growing at an unprecedented rate. The capability of HAlign4 to efficiently handle millions of sequences not only saves time but also provides an accessible solution for laboratories with limited computational resources. This scalability ensures that researchers can conduct comprehensive genomic analyses without the bottlenecks often associated with memory and processing limitations.

Beyond its computational advantages, HAlign4 maintains high alignment accuracy, comparable to leading alignment tools such as MAFFT, while outperforming others like MUSCLE and ClustalΩ, which fails to complete alignments for longer sequences. The alignment quality of HAlign4 is particularly notable for maintaining consistency across a wide range of sequence similarities and lengths, making it suitable for diverse applications, from viral genome tracking to evolutionary studies. The combination of speed, memory efficiency, and alignment accuracy positions HAlign4 as a versatile and powerful tool for multiple sequence alignment, especially suited for large and complex datasets. The incorporation of advanced data structures and alignment algorithms not only enhances its efficiency but also ensures that the alignment process remains reliable, even under challenging conditions involving high sequence variability and length heterogeneity.

In addition to its core features, HAlign4 provides a user-friendly interface and supports parallel computing, enabling users to fully leverage multi-core processors for faster alignment. This feature is particularly beneficial in environments where time is a critical factor, such as during pandemic responses or large-scale biodiversity assessments. The combination of advanced algorithmic techniques and practical usability makes HAlign4 an ideal choice for bioinformaticians seeking a reliable tool that can adapt to both small-scale and large-scale projects. Furthermore, the open-source nature of HAlign4 encourages community-driven improvements and adaptations, fostering a collaborative environment where the tool can evolve in response to emerging needs and challenges in the field.

In summary, HAlign4 represents a significant improvement in multiple sequence alignment methodologies, effectively addressing the key limitations of prior approaches and offering a reliable solution for modern bioinformatics challenges. Its ability to balance speed, accuracy, and resource efficiency makes it a valuable asset for researchers working with increasingly large and complex datasets. Future work may focus on incorporating clustering algorithms and post-alignment processing strategies to further enhance the quality of star alignments. In addition, exploring the integration of machine learning techniques could provide further optimization opportunities, enabling adaptive alignment strategies that respond dynamically to the specific characteristics of the input data. Such advancements would further solidify HAlign4’s position as a leading tool in the rapidly evolving landscape of bioinformatics.
